# Missed Opportunities: Barriers to HIV Testing during Pregnancy from a Population Based Cohort Study in Rural Uganda

**DOI:** 10.1371/journal.pone.0037590

**Published:** 2012-08-16

**Authors:** Elin C. Larsson, Anna Ekéus Thorson, George Pariyo, Peter Waiswa, Daniel Kadobera, Gaetano Marrone, Anna Mia Ekström

**Affiliations:** 1 Division of Global Health, Department of Public Health Sciences, Karolinska Institutet, Stockholm, Sweden; 2 Makerere University School of Public Health, Kampala, Uganda; 3 Iganga/Mayuge Health and Demographic Surveillance Site, Iganga, Uganda; University of Buea, Cameroon

## Abstract

The aim was to assess population-level HIV-testing uptake among pregnant women, key for access to prevention-of mother to child transmission (PMTCT) services, and to identify risk factors for not being HIV tested,

The study was conducted May 2008–May 2010 in the Iganga/Mayuge Health and Demographic Surveillance Site (HDSS), Eastern Uganda, during regular surveillance of 68,000 individuals. All women identified to be pregnant May–July 2008 (n = 881) were interviewed about pregnancy-related issues and linked to the HDSS database for socio-demographic data. Women were followed-up via antenatal care (ANC) register reviews at the health facilities to collect data related to ANC services received, including HIV testing. Adjusted relative risk (aRR), and 95% confidence intervals (CI) for not being HIV tested were calculated using multivariable binomial regression among the 544 women who remained after record review.

Despite high ANC attendance (96%), the coverage of HIV testing was 64%. Only 6% of pregnant women who sought ANC at a facility without HIV testing services were referred for testing and only 20% received counseling regarding HIV. At ANC facilities with HIV testing services, 85% were tested. Only 4% of the women tested had been couple tested for HIV. Living more than three kilometers away from a health facility with HIV testing services was associated with not being tested both among the poorest (aRR,CI; 1.44,1.02–2.04) and the least poor women (aRR,CI;1.72,1.12–2.63).

The lack of onsite HIV testing services and distant ANC facilities lead to missed opportunities for PMTCT, especially for the poorest women. Referral systems for HIV testing need to be improved and testing should be expanded to lower level health facilities. This is in order to ensure that the policy of HIV testing during pregnancy is implemented more effectively and that testing is accessible for all.

## Introduction

An estimated 17,000 children in Uganda were infected with HIV through mother-to-child transmission (MTCT) in 2009, despite a stable HIV prevalence of 7.5% among women of reproductive age (15–49 years) and high (94%) uptake of antenatal care (ANC) [1], [2]. HIV testing is the first and crucial step in prevention of MTCT (PMTCT); however, only 63% of pregnant Ugandan women were tested in 2010 [3]. A reduction in MTCT rate from roughly 40% to around 1% is possible through the provision of prophylactic therapy with three antiretroviral medicines (ARVs) administered to HIV infected women before, during and after delivery, cesarean section, and provision of prophylactic ARVs to newborns and replacement infant feeding [4]. In low-income countries with limited access to combination ARV regimens or the capacity to provide cesarean section and replacement feeding, the rate of MTCT can still be reduced to around 5%, provided that women are tested, enrolled in and complete the PMTCT program [5]–[7].

Long waiting times at health facilities and lack of comprehensive information about HIV and PMTCT, spousal disapproval and stigma, are generally suggested as barriers to the uptake and completion of PMTCT [8]–[11]. The World Health Organization (WHO) recommends that countries with generalized HIV epidemics implement opt-out or provider-initiated HIV testing during ANC to increase likelihood of HIV testing among pregnant women [12]. Health facility-based studies have indicated an increase in HIV testing rates during pregnancy and thus support utilization of these recommendations [13]–[18]. Accordingly, the Ugandan national policy guidelines for PMTCT recommends that all pregnant women are counseled and tested for HIV during ANC, provided they do not actively opt-out, or referred for testing if onsite services are not available [19]. Additionally, but so far with limited success in practice, joint couple HIV testing including the pregnant woman and her spouse has also been introduced in Uganda, hoping to expand the coverage of HIV testing and to reduce barriers to accessing PMTCT services [20]–[24].

Despite these efforts at the national level, evidence on the effectiveness of policy implementation at population-level is still lacking. This study examines barriers to HIV testing in the context of the national opt-out HIV testing policy and assesses population-based coverage of HIV testing during pregnancy in a prospective cohort of pregnant women identified through a health and demographic surveillance site in Uganda.

## Methods

### Study setting

This population-based cohort study was conducted between May 2008 and May 2010 at the Iganga/Mayuge Health & Demographic Surveillance Site (HDSS), a designated area across parts of the Iganga and Mayuge districts in eastern Uganda. The HDSS is predominantly rural, but also partly semi-urban (Iganga Town). The main source of income in the area is subsistence farming [25]. In 2008, the HDSS population was 68,000 individuals among 12,000 households. HDSS activities include data collection on births, deaths, pregnancies, and migration three times per year and have been described elsewhere [26]. Add-on surveys for special studies, such as part of this study, are also conducted between or during regular data collection rounds.

The estimated HIV prevalence among Busoga women aged 15–49 years is 5.6%, which, for this regionally dominant ethnic group, is lower than the Ugandan average for women of reproductive age (7.5%) [27]. Ugandan health facilities range from the lowest level II health centres (midwife available) to hospital level (level V). ANC is provided at all levels and should include a physical examination, the provision of necessary medicines, health education, and HIV counseling and testing, or HIV testing referral. The Ugandan national guidelines recommend at least four ANC visits per pregnancy [28]. In the HDSS area, 13 health facilities provided ANC, and one-third of these offered PMTCT services, a proportion corresponding to the overall accessibility to PMTCT services across the two districts. Very few other options for HIV testing existed in this area at the time of study and to our knowledge, no home-based HIV testing or out-reach testing was carried out.

At the time of enrolment for this study, WHO recommended the use of dual ARV prophylaxis of zidovudine (AZT) and lamivudine (3TC) from pregnancy week 28 [29]. The ARV regimen used at lower level health facilities in the two districts was single dose nevirapine (sd-NVP), while the hospital provided either sd-NVP or the dual ARV regimen, or triple ARVs for women eligible for that for her own health. Most of the HIV-infected women in this setting received a dual combination ARV prophylaxis.

### Study design and data collection

Ethical approval was obtained from Makerere University School of Public Health Institutional Review Board and the Uganda National Council for Science and Technology (Ref.nr. HDREC, 052). The HDSS setup was used to determine the population coverage of HIV testing. All women in the 12 000 households who were pregnant (n = 881) at the time of study enrollment May–July 2008 were identified through self-reports as part of routine data collection on pregnancies in the HDSS. Within 2–6 weeks after this screening, field-assistants visited these women at their homes and after describing the purpose and specifics of study, invited the women to participate in a interview. All women agreed. Written informed consent was obtained from all participants at this point of enrolment, which included consent to link women's interview data to health facility and core HDSS data.

Four field-assistants (females due to the sensitive nature of some questions related to women's health and pregnancy) were trained for three days. The training focused on how to ask questions about pregnancy and HIV and how to use the questionnaire, which included questions about personal characteristics, ANC attendance and care received. The questionnaire was translated into the local language, Lusoga, piloted and adjusted to clarify some minor issues. During data collection, a field-supervisor held daily meetings with the field-assistants to deal with any problems arising.

At enrolment, 412 (47%) of the 881 women had not yet attended ANC during the current pregnancy, most of them because they were in an early gestational stage. These 412 women were therefore followed up for second interview after their expected delivery (interviewed May–August 2009) to capture any information regarding ANC attendance. The follow up interviews covered the same questions as those put to the women who had sought ANC already at enrolment in 2008. Out of these 412 women, 279 (68%) could be followed up and of these 41 women had not sought ANC at all (31 because they had had a miscarriage and l0 declined to give any reason). The 133 women that could not be followed up had either moved or were otherwise unavailable (some were not at home during any of the three study visits) and therefore excluded from the study. Hence, a total of 707 women (881-41-133) were followed up through ANC record reviews at the health facilities where they had sought ANC, to collect data on type of ANC received, including HIV testing. The first author and two trained field-assistants with ANC experience carried out the record reviews (in February–May 2010).

Identification was confirmed by the use of available HDSS information on name, age, expected time of delivery, home village and name of spouse. Interview and record data were thereafter linked to core HDSS socio-demographic data and distances to health facilities for each respondent.

### Data

Data were double entered in separate entry forms in SPSS software [30], merged using STATA software [31], and linked to demographic data from the HDSS database using the women's HDSS identity numbers.

The outcome under study was: “not being tested for HIV during current pregnancy” (obtained from record reviews). Based on existing literature, various potential risk factors for not being tested for HIV were then identified, and preliminarily divided: (i) socio-demographic characteristics (ii) health-seeking behavior (iii) health facility-related factors and (iv) distance to health facilities (GPS data).

Socio-demographic characteristics included age, occupation, household size, number of living children, spousal support and socioeconomic status (SES). The HDSS keeps asset-based SES data on each household as wealth quartiles generated from principal components analysis [32], [33] and asset variables identified by the Uganda Bureau of Statistics, which have been described in detail elsewhere [26]. We first examined wealth quartiles and the two lowest quartiles were then grouped into “poorest women”, and the highest two into “least poor women”, due to similar associations with not being HIV tested for these categories. Age was grouped into <21, 21–34, and >34 years, since young and older women would have different childbirth experiences. Occupation was categorized into ‘farmers versus other’ and number of living children into 0–4 children versus >4, based on a median number of 4 children.

Da ta on health-seeking behavior were defined as adherence to national guidelines for ANC, type of facility where women sought ANC, whether anyone advised the woman to seek ANC. Seeking ANC late was defined as attending ANC after the sixth gestational month.

The shortest distance from home to the nearest ANC health facility and to the nearest facility with onsite HIV testing, was estimated using GPS coordinates. Distance was first categorized into three equally large groups; 0–1; 2–3, and >3 kilometers, but the first two categories were later merged when analyzing “distance to nearest facility with onsite HIV testing”, due to their similar associations with not being HIV tested.

During an initial descriptive analysis, chi-square statistics were used to compare the relationship between the risk factors and not being tested for HIV. Though we grouped the factors as described above, their association with the outcome was independently assessed, first by calculating bivariate relative risk estimates, using binomial regression models with link log. Independent variables with a p-value<0.25 in bivariate analysis were tested in the multivariable model. By using a backward selection procedure we created a final multivariable model that included SES, number of children and distance to a health facility with onsite HIV testing.

All the variables in the final model were tested for interaction and the only statistically significant was between the variables “number of children” and SES. Therefore we stratified the data analysis by SES to account for effect modification.

In the multivariable models variables were considered statistically significant if the adjusted p-value was <0.05.

## Results

### Uptake of ANC and HIV testing

**Figure 1:**
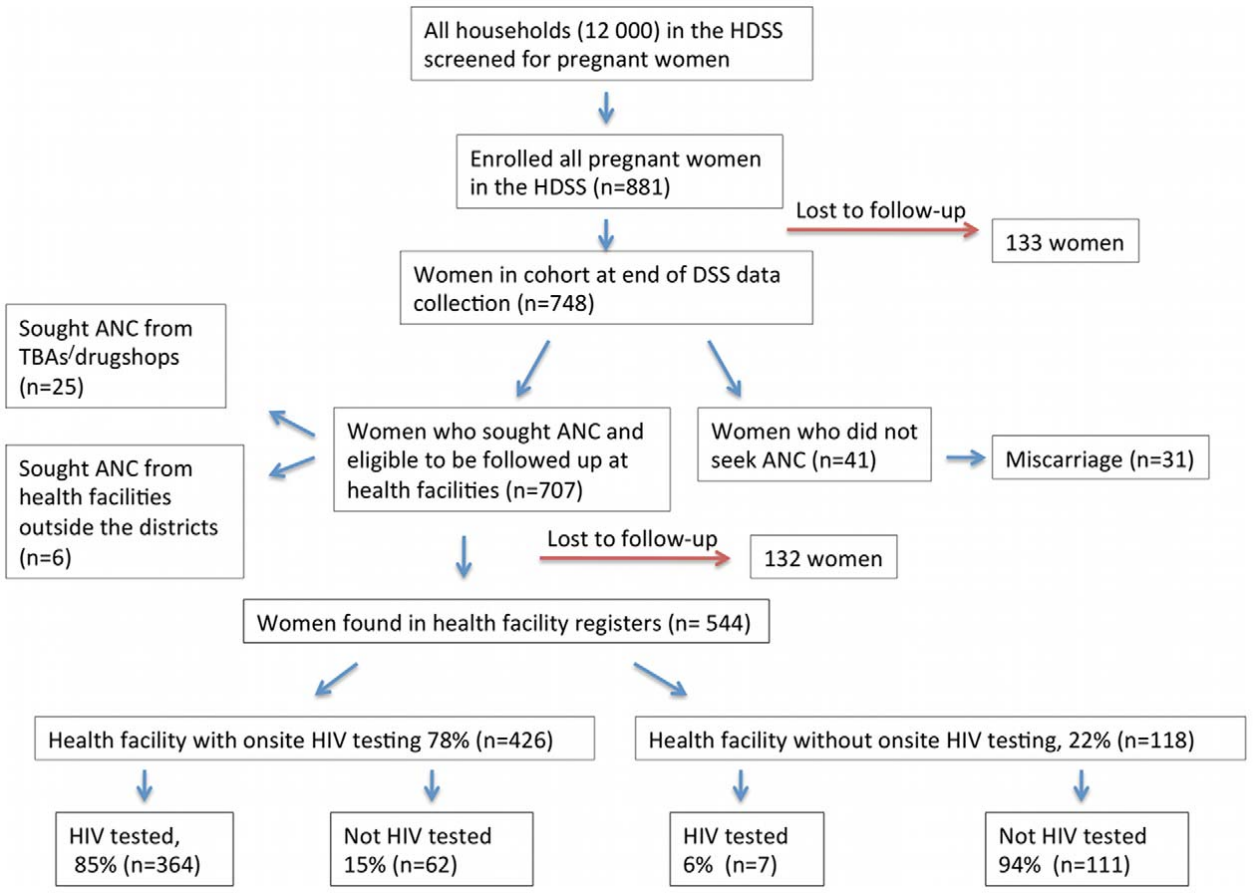
Flow of study participants: pregnant women enrolled, seeking ANC and being tested for HIV.

**Figure 2:**
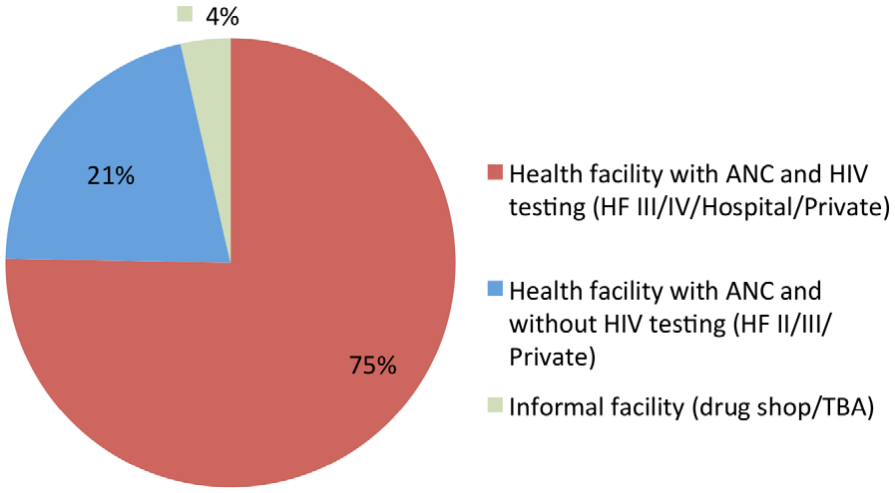
Type of health facility where the women sought ANC.

Figures 1 and 2 depict the flow of study participants and show the percentages of those women who sought ANC and received HIV testing. Of the 707 women who reported they sought ANC 25 (4%) attended ANC from a traditional birth attendant (TBA) or at a drug-shop, hereinafter termed “informal facility”, hence 682 women (96%) sought ANC at least once. Four hundred and twenty-six (78%) out of 682 received ANC at a health facility that offered onsite HIV testing, while 118 women (22%) attended a health facility that did not offer onsite HIV testing. The majority (n = 544; 80%) of the women reporting ANC visits could also be traced back to the ANC registers. Overall, only 64% (371/579) of the women were HIV tested during the current pregnancy (the denominator of 579 includes 544 found in registers +10 who did not seek any ANC at all+25 who sought ANC at informal facilities, Fig. 1).

### ANC and HIV testing by health facility

**Figure 3:**
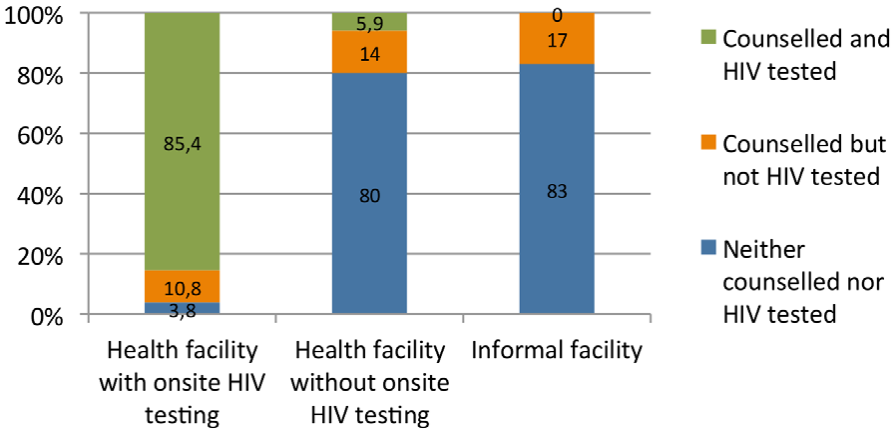
Percentages of pregnant women counseled and tested for HIV by type of health facility.

Figure 3 shows the proportion of women tested for HIV by the type of health facility. The majority of the women who attended a health facility that offered onsite HIV testing, 85%, were counseled and HIV tested. However, only 20% vs. 17% of women who attended health facilities that did not have HIV testing onsite vs. informal facilities, respectively, were counseled about or counseled and tested for HIV. Only 6% of pregnant women who initially went for ANC at a health facility that did not have onsite HIV testing were HIV tested, either because they were not referred by the staff or because they had not followed-up on the recommendation to test elsewhere.

### Sample characteristics and health-seeking behavior and distances to health facilities

Table 1 presents the sample characteristics and health-seeking behaviors among the women. About half of the women who sought ANC did so at hospital-level (50.1%). Although 96% did at least one ANC visit, only 28% fulfilled the three ANC visits. Using GPS coordinates, about one-third (30.7%) had to travel four kilometers or more to access a facility that offered onsite HIV testing. Very few male partners attended ANC with their wives (12.7%). The record reviews performed at the ANC health facilities that provided HIV testing showed that the uptake of couple testing was only 4% among women who tested for HIV.

**Table 1:** Socio-demographic factors, health-seeking behavior and likelihood of not testing for HIV during pregnancy among women seeking ANC (n = 707).

Characteristic	All women, n (%)	Not tested for HIV, n* (%)	Tested for HIV, n* (%)	Crude RR** (95%CI)	aRR (95%CI)	
Demographic information	Column %	Row %	Row %		Poorest^b^	Least poor^b^
Age	Median: 25.5 Range: 14–50	Median: 25.7	Median: 24.7			
14–20	140 (19.2)	34 (34.7)	64 (65.3)	1		
21–34	510 (70.1)	115 (31.8)	247 (68.2)	0.92 (0.67–1.25		
35+	78 (10.7)	18 (39.1)	28(60.9)	1.13 (0.73–1.75)		
Number of people living in the household						
1–5	199 (30.9)	69 (32.9)	141 (67.1)	1		
6–9	324 (50.2)	53 (35.0)	96 (64.4)	1.07		
10+	122 (18.9)	29 (35.4)	53 (64.6)	1.08 (0.74–1.59)		
Occupation						
Subsistent farmer	511 (73.0)	146 (37.2)	247 (62.8)	1		
All other	189 (27.0)	27 (17.9)	124 (82.1)	2.08 (1.36–3.18)		
Socio-economic status^a^						
Least poor	283 (49.7)	57 (29.2)	138 (70.8)	1		
Poorest	287 (50.3)	81 (40.7)	118 (59.3)	1.39 (1.05–1.84)		
Number of living children	Median 4.0 Range: 1–11					
0–4	433 (80.2)	122 (28.2)	311 (71.8)	1	1	1
5+	107 (19.8)	47 (43.9)	60 (56.1)	1.56 (1.21–2.00)	1.03 (0.64–1.67)	1.77 (1.00–3.16)
Mean of transport to ANC						
By foot	304 (43.0)	98 (42.1)	135 (57.9)	1.74 (1.35–2.25)		
Other means	403 (57.0)	75 (24.1)	236 (75.9)	1		
Partner attended ANC during current pregnancy						
Yes	90 (12.7)	15 (23.1)	50 (76.9)	1.22 (0.74–2.01)		
No	617 (87.3)	158 (33.0)	321 (67.0)	1		
**Health-seeking behavior**						
Person advising woman to seek ANC						
Nobody	587 (83.0)	139 (30.3)	320 (69.7)	1		
Spouse	62 (8.8)	18 (40.9)	26 (59.1)	1.35 (0.94–1.94)		
Mother/Mother in-law	43 (6.1)	8 (26.7)	22 (73.3)	0.88 (0.47–1.66)		
Other	15 (2.1)	8 (72.7)	3 (27.3)	2.40 (1.60–3.59)		
Number of ANC visits during current pregnancy	Median: 2 Range: 1–6					
1	206 (38.1)	56 (27.2)	150 (72.8)	1		
2	181 (33.4)	60 (33.1)	121 (66.9)	1.22 (0.90–1.66)		
3+	154 (28.5)	54 (35.1)	100 (64.9)	1.29 (0.94–1.76)		
Seeking ANC the first time after month 7						
Yes	219 (44.2)	67 (30.6)	152 (69.4)	1		
No	276 (55.8)	67 (24.3)	209 (75.7)	1.39 (1.00–1.94)		
Level of care where ANC was sought						
Hospital	350 (50.1)	33 (12.1)	239 (87.9)	1		
Level III/Level IV	184 (26.3)	49 (32.0)	104 (68.0)	2.64 (1.71–4.06)		
Private clinic/Level II	165 (23.6)	91 (76.5)	28 (23.5)	6.30 (4.24–9.37)		
Seeking ANC from a health facility without onsite HIV testing services						
Yes	173 (24.7)	111 (94.1)	7 (5.9)	6.46 (5.16–8.10)		
No	527 (75.3)	62 (14.6)	364 (85.4)	1		
Amount paid for ANC (USD)						
0	253 (31.2)	42 (39.2)	65 (60.8)	1		
0.1–1	340 (41.9)	86 (31.2)	190 (68.8)	1.40 (1.00–1.98)		
1-max (11.7)	219 (27.0)	45 (27.9)	116 (72.1)	1.11 (0.82–1.52)		
Received counseling for HIV testing (self-reported)						
Yes	484 (68.6)	60 (15.2)	334 (84.8)	1		
No	222 (31.4)	113 (75.3)	37 (24.7)	4.95 (3.81–6.42)		
**Distance**						
Distance to health facility where woman sought ANC	Range: 0–4 km					
0–1 km	66 (11.4)	60 (33.2)	121 (66.8)	1.65 (1.13–2.41)		
1–3 km	321 (55.3)	27 (19.7)	110 (80.3)	0.887 (0.63–1.26)		
4+ km	194 (33.4)	39 (26.9)	106 (73.1)	1		
Distance to nearest health facility with HIV testing	Range: 0.12–6.7 km					
0–3 km	461 (69.3)	94(25.9)	269 (74.1)	1	1	1
4+ km	204 (30.7)	69 (45.4)	83 (54.6)	2.57 (1.36–4.86)	1.44 (1.02–2.04)	1.72 (1.12–2.63)

a Calculated using principle component analysis on household assets divided into two groups.

b Adjusting for age.

* n = 544 with information on HIV testing.

** Crude RRs shown only for variables with p-value<0.25.

Women who had not been HIV tested had an average distance of 3.5 kilometers to the nearest testing facility, compared to 2.8 kilometers among women who had been tested (t-test, p<0.01).

Moreover, the poorest women had 3.5 kilometers to a health facility with testing services as compared to 2.7 km for the least poor (t-test, p<0.01).

### Risk factors for not being tested for HIV during pregnancy

The bivariate analysis identified several risk factors (p<0.25) for not being tested for HIV: being a farmer, being poor, having more than four living children, living more than 3 kilometers from a facility with onsite HIV testing, not seeking ANC at one's own initiative, seeking ANC late (first visit later than gestational month six) and, somewhat surprisingly but inversely related to seeking ANC late (Table 1). Table 1 also presents other potential risk factors that were analyzed but that was shown to be non-significant (p>0.25) and not analyzed in the multivariable model.

The risk factors (p<0.25) were then analyzed in a multivariable model using a backwards selection procedure. The multivariable analysis showed that when stratified by income level, the only significant risk factor for not being tested for HIV among the poorest women was living more than three kilometers away from a health facility with testing services (aRR 1.44, 95% CI 1.02–2.04), adjusting for age (Table 1). Among the least poor, having more than four children (aRR 1.77, 95% CI 1.00–3.16), and living more than three kilometers from a health facility with testing services (aRR 1.72, 95% CI 1.12–2.63), were significant predictors of not being tested, after adjusting for age (Table 1).

For the women who attended ANC at a facility without HIV testing, but who reported that they had been counseled for HIV testing, 13% were HIV tested, as compared to 4% among women who said they had not been counseled.

## Discussion

This population-based study from two rural Ugandan districts found that the uptake of HIV testing among pregnant women was 64% ,i.e. very similar to the WHO 2010 estimate of 63% testing coverage at national level [3]. The study also provides further insight into why less than two-thirds of pregnant women ever pass the key entry-point to access PMTCT. Apparently, non-referral for HIV testing from more distant health facilities that lack HIV testing onsite poses a major bottleneck for increased PMTCT coverage, especially for the poorest women and despite higher than national average levels of ANC attendance in this setting 96% vs. 94%[2].

The opt-out testing policy seems to work fairly well in facilities where HIV testing is available onsite since 85% of the women who attended such facilities were tested. This finding is also supported by previous research [34]. However, very few women, only 6% who attended ANC at health facilities without testing onsite, were referred or carried out referral for testing. Moreover, only 20% of the women seeking ANC at facilities without testing had been counseled about or counseled and tested for HIV; and yet, the Ugandan PMTCT guidelines state that all pregnant women should be counseled [19]. This finding is consistent with a recent qualitative study conducted from the same area which suggested inadequate HIV counseling and a lack of testing referral from this type of facility [10]. Our analysis further reports that male partner support was low, only 13% of the pregnant women reported being accompanied to the ANC facility by their spouse, and 4% of the women had had couple HIV testing.

Previous findings from the same setting suggest a combination of health system failures and traditional gender structures as important barriers to male partner involvement [20], [22]. Firstly, men are recruited for couple HIV testing through their pregnant spouse who are told by the midwife to fulfill the challenging, and often impossible task to convince the male family decision-maker to go for couple testing [20], especially for women in unstable relationships Secondly, men are less likely in general to seek any type of health care and especially ANC, which is viewed as a female domain [22]. For more successful policy-implementation and involvement of male partners in ANC and PMTCT, knowledge about local gender structures are needed, including a direct targeting of men at their own conditions.

Poverty has also previously been reported as an important barrier to PMTCT services in Uganda [35]. Thus, although inequity in access to health care has improved dramatically since the 1970s, when it was acknowledged at global level, much remains to be done in terms of poor people's right to basic health care and before universal access could become more realistic [36], [37].

The increased risk for not being HIV tested among women with many children corresponds to a previous finding in the same setting indicating that women who are tested during a previous pregnancy feel less motivated to test again [38]. Nevertheless, failure to test for HIV during all pregnancies could have important implications for MTCT in Uganda, which has one of the highest fertility rates in the world (6.7) as well as a high prevalence of concurrent partnerships [39]. Our population-based findings differ from those from a hospital-based study, also in eastern Uganda, where having more children was associated with a positive attitude towards testing during ANC [34]. Having many children might also be confounded by educational level or staff attitudes towards the benefit of PMTCT, factors not analyzed here.

The known inverse relationship between distance to a health facility and health care seeking played an important role also for HIV-testing in this setting where a previous study also showed that living three kilometers or more from a health facility decreased the likelihood of seeking facility care for febrile children [40]. The Ugandan National Health Policy recommends that all people should have a maximum distance of five kilometers to a health facility, and reducing that distance to three kilometers, the cutoff barrier identified in our study for seeking adequate care, appears unrealistic [41]. Thus, to improve effective PMTCT coverage in Uganda, referral systems for HIV testing must improve, in parallel with an expansion of onsite and/or out-reach HIV testing to lower level facilities as part of routine care. Scaled-up out-reach testing may be preferable due to human resource shortages in Uganda and the risk of insufficient quality of counseling as PMTCT services are expanded to lower levels of care with inadequately trained staff [42]. However, the feasibility of testing at lower-level facilities may be enhanced if accompanied by community-based peer-counseling [43].

The low coverage of HIV testing and the above discussion clearly illustrates the Millennium Development Goal Countdown discourse on the importance of well-functioning health systems to successfully implement large-scale interventions, such as PMTCT [36].

None of the eligible women declined participation, possibly due to the fact that the HDSS collect data regularly, making women used to responding to questions and possibly more keen to participate in research. The women lost to follow-up did not differ significantly from the responders in terms of socio-demographic baseline information. The loss of respondents at health facility level was due random mistakes by the midwives and evenly distributed among the facilities, thus should not have biased the results. For the record reviews we used the HDSS information described in the method section and when uncertain about identification, the woman was excluded. We relied on register data for some variables, and must therefore contend with the possibility that what is defined as lacking in receipt of certain care aspects, might in some cases be due to mis- or unregistered information. However, most registers appeared to be completed appropriately for the selected variables, especially for HIV testing.

## Conclusions

High ANC attendance, yet low HIV testing coverage, demonstrates missed opportunities to enroll women into PMTCT services. This study shows, at population-level, that lack of onsite HIV testing services, poverty, failure to adjust to existing gender norms and distance to health facilities are clear barriers to effective policy implementation and high HIV testing uptake. The health system in terms of policies, funds, and human resources, needs to be mobilized to ensure that HIV testing is available and accessible for all pregnant women during ANC. Without increasing the uptake of this first and crucial component of the PMTCT program, the UNAIDS call for “Virtual elimination of HIV infections due to MTCT” is unachievable.
